# Cancer drug sensitivity prediction from routine histology images

**DOI:** 10.1038/s41698-023-00491-9

**Published:** 2024-01-06

**Authors:** Muhammad Dawood, Quoc Dang Vu, Lawrence S. Young, Kim Branson, Louise Jones, Nasir Rajpoot, Fayyaz ul Amir Afsar Minhas

**Affiliations:** 1 Tissue Image Analytics Centre, University of Warwick, Coventry, UK; 2 Warwick Medical School, University of Warwick, Coventry, UK; 3 Cancer Research Centre, University of Warwick, Coventry, UK; 4 Artificial Intelligence & Machine Learning, GlaxoSmithKline, San Francisco, CA USA; 5 Barts Cancer Institute, Queen Mary University of London, London, UK; 6 The Alan Turing Institute, London, UK

**Keywords:** Breast cancer, Breast cancer

## Abstract

Drug sensitivity prediction models can aid in personalising cancer therapy, biomarker discovery, and drug design. Such models require survival data from randomised controlled trials which can be time consuming and expensive. In this proof-of-concept study, we demonstrate for the first time that deep learning can link histological patterns in whole slide images (WSIs) of Haematoxylin & Eosin (H&E) stained breast cancer sections with drug sensitivities inferred from cell lines. We employ patient-wise drug sensitivities imputed from gene expression-based mapping of drug effects on cancer cell lines to train a deep learning model that predicts patients’ sensitivity to multiple drugs from WSIs. We show that it is possible to use routine WSIs to predict the drug sensitivity profile of a cancer patient for a number of approved and experimental drugs. We also show that the proposed approach can identify cellular and histological patterns associated with drug sensitivity profiles of cancer patients.

## Introduction

The premise of precision medicine is to develop therapies that target important characteristics such as the molecular profile of an individual tumour. The outcome of drug therapy is often unpredictable ranging from desirable to toxic and predominantly driven by a tumour’s molecular profile^1^. The response to anti-cancer drugs can be influenced by both germline and acquired somatic mutations^2^ as well as the status of molecular/signalling pathways^3^, suggesting that therapies targeting the genomic landscape of an individual are more effective compared to one-size-fits-all therapy approaches^4^. Pharmacogenomics is a pivotal component of precision oncology that fuses pharmacology and genomics to study an individual’s response to drug based on their genomic profile^5^. Recent advances in high-throughput drug screening and the availability of pharmacological data together with a multitude of omics data (genomic, mutational, transcriptomic, proteomic and metabolomic data) have paved the way for identifying genetic biomarkers that are associated with treatment response^6,7^.

Cancer cell lines (CCLs) provide an easy-to-manipulate vehicle for high-throughput drug screening at scale, prior to the more expensive in vivo testing and clinical trials of a drug^6^. Pioneers of these large-scale genomic and drug screening datasets include the NCI-60 database^8^, Cancer Cell Line Encyclopedia (CCLE)^9^, Genomics of Drug Sensitivity in Cancer (GDSC)^10^, and the Cancer Therapeutic Response Portal (CTRP)^11^. These datasets have helped assess the sensitivity of many compounds including FDA-approved drugs in vitro and have led to the discovery of novel anti-cancer therapies^12,13^. The pharmacogenomics data provided by these initiatives have enabled collective analysis of drug sensitivity and gene expression data to uncover novel drug–gene relationships^8^. Several machine learning (ML) methods have been proposed for associating mutation and gene expression data of CCLs with their respective drug efficacy metrics such as half maximal inhibitory concentration (IC_50_), or area under the dose-response curve (AUC-DRC)^10,14,15^. Similarly, deep learning (DL) based methods^16–19^ and several other approaches^20–22^ have been proposed for predicting patient response to drugs using genomic information.

Despite advances in genomics-based drug sensitivity analysis, the applicability of genomics profiling for selecting appropriate drugs remains limited. The digitisation of tissue slides and recent advances in detailed tissue profiling using digital scans of routine Haematoxylin and Eosin (H&E) stained tissue slides offer a new way to predict drug sensitivity via spatial histological profiling. To the best of our knowledge, this is the first study that proposes the prediction of patients’ sensitivity to multiple drugs from routine H&E images by training a predictive model using drug sensitivity data imputed from CCLs. We address the question as to whether and to what extent it is possible to predict a breast cancer patient tumour’s sensitivity to multiple approved and experimental drugs based on their histological profile as captured by DL-based analysis of the H&E images. The resulting association of visual histological patterns with drug sensitivity can be helpful in identifying histological motifs associated with high and low sensitivity of drugs. Not only can it pave the way for spatial characterisation of treatment response, but it also carries the potential of ruling out treating a patient with certain drugs due to their histological profile.

Histological examination of tissue sections is considered a gold standard for the clinical diagnosis of solid tumours. Recent advancements in deep learning for computational pathology have proven valuable for using WSIs of routine H&E-stained tissue sections to predict cancer subtypes^23,24^, patient survival^25,26^, mitosis detection^27^, DNA methylation patterns^28^, cellular composition^29,30^, and tumour mutation burden^31^. Moreover, histology image-based prediction of mutation and expression profile of different genes^32–34^ and prediction of molecular markers and pathways^35,36^ has been achieved using DL. Recently, a DL model has been proposed for predicting breast cancer patients gene expression state from WSIs^37^.

**Fig. 1:**
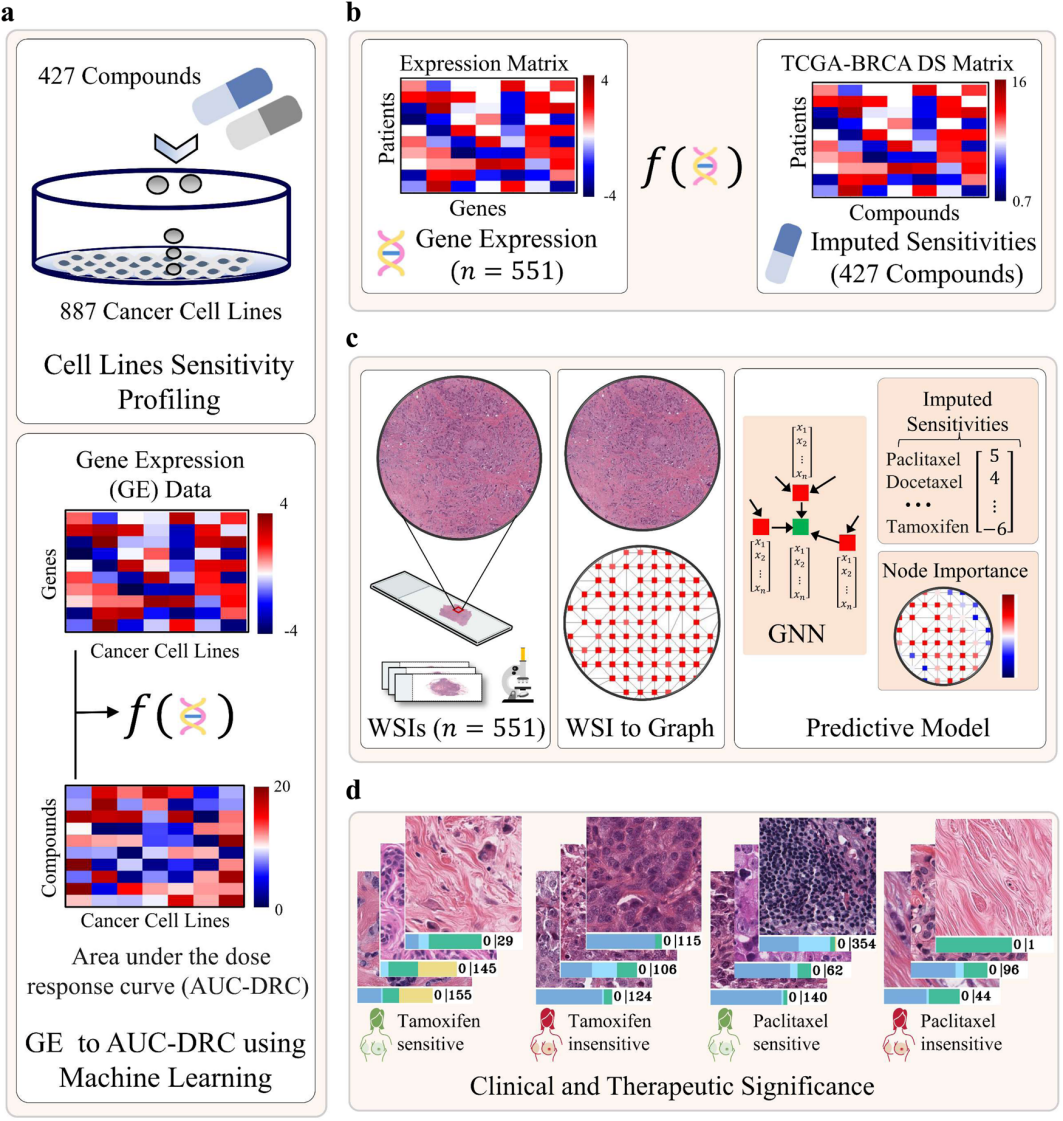
Workflow of the proposed approach for predicting patient sensitivities to different drugs from histology images. **a** Regression model was developed using cancer cell line gene expression data and in vitro drug screening data to learn the association between cell lines gene expression profile and their sensitivity to 427 compounds. **b** The trained model was then used to infer the sensitivity of breast cancer patients to these drugs based on their gene expression. The output of the model is a matrix listing the gene expression-based imputed drug sensitivities of each patient (one per row) to 427 compounds (one per column). **c** Prediction of patient sensitivities to compounds from whole slide images (WSIs) of formalin-fixed paraffin-embedded (FFPE) H&E-stained tissue section using a Graph Neural Network (GNN). We represent each WSI as a graph and then pass the WSI-graph as input to a GNN for predicting WSI-level and patch-level sensitivities of a patient to different drugs. Node-level prediction highlight the spatially resolved contribution of different region of WSI towards the predicted sensitivity of a certain drug. **d** Histological motifs associated with high and low sensitivity of Tamoxifen and Paclitaxel are shown for illustration.

In this work, we investigate the association between cellular and morphometric patterns contained in the digitised WSIs of routine H&E tissue slides of breast cancer tumours and their drug sensitivity profiles. We employ the method proposed in^37^ for predicting patient’s likelihood of response to treatment to specific drugs. The framework proposed in this study (see Fig. 1) offers several possible advantages. First, it enables a direct association of phenotypic information present in WSIs with the likelihood of response to different drugs. Second, by utilising pharmacogenomic datasets, a large population of patients can be virtually screened for a broad spectrum of compounds in a relatively short amount of time giving valuable insights into the association of patient-specific histological signatures with drug sensitivities. Third, harnessing ML for drug sensitivity estimates allows modelling the relative contribution of the expression level of various genes to the patient’s sensitivity to a broad spectrum of compounds in an unbiased manner. The proposed framework does not rely on any assumption about the mechanism of action of compounds, which may be unknown, or on patient’s survival data. Fourth, the framework offers flexibility to investigators when studying the response of a lead compound in different tissue types, subtypes, or any other patient population as part of a drug discovery pipeline. Finally, histological patterns discovered using the proposed framework can be easily translated into clinical practice as it is built on digital scans of routine H&E tissue slides and does not require any expensive or time-consuming assays.

## Results

### Imputed drug sensitivities from cell lines

We used estimated sensitivities of the Cancer Genome Atlas breast cancer (TCGA-BRCA) patients to 427 compounds from a previously published method^15^, which fits a linear ridge regression model between CCLs based gene expression data of genes as input and the corresponding in vitro measurement of drug response in terms of AUC-DRC as output. Once trained, these linear regression models (one per drug) were then used for imputing the sensitivity of TCGA-BRCA patients to every drug in the CTRP database^11^. An overview of the approach used for obtaining patients ground-truth sensitivity estimates is provided in Fig. 1a, b. It is important to note that the higher the AUC-DRC, the lower the drug sensitivity since a higher concentration of the drug is required for it to be effective and vice versa.

### Analytical pipeline for whole slide image analysis and predictive modelling

To explore the association between cellular and histological patterns contained in WSIs and patient tumour’s sensitivity to different drugs, we propose an end-to-end DL pipeline that takes WSI of a patient as input and predicts the sensitivity of 427 compounds as output. An overview of the proposed framework is provided in Fig. 1c. We employed our in-house ${{SlideGraph}}^{\infty }$ pipeline^37^ that first constructs a graph representation of the WSI and then uses a graph neural network (GNN) to predict node-level (patch-level) and WSI-level sensitivity of a patient to all the compounds (compounds listed in Supplementary Data 1). The node-level scores are then used to identify regions within the WSI that contribute to high or low sensitivity. This gives insight into different types of cells present in the tumour microenvironment (TME) in a spatially resolved manner as shown in Fig. 1c, d. In this study, we utilised WSIs of TCGA-BRCA patients (*n* = 551) that have gene expression-based imputed sensitivity score for all 427 drugs. Finally, we developed an interactive web interface called Histology image-based Drugs Sensitivity Prediction (HiDS), that allows users to analyse spatially resolved contribution of different regions of the WSI towards predicted sensitivity estimates. The interactive web interface can be accessed at (http://tiademos.dcs.warwick.ac.uk/bokeh_app?demo=HiDS).

### Prediction of drug sensitivity from whole slide images

**Fig. 2:**
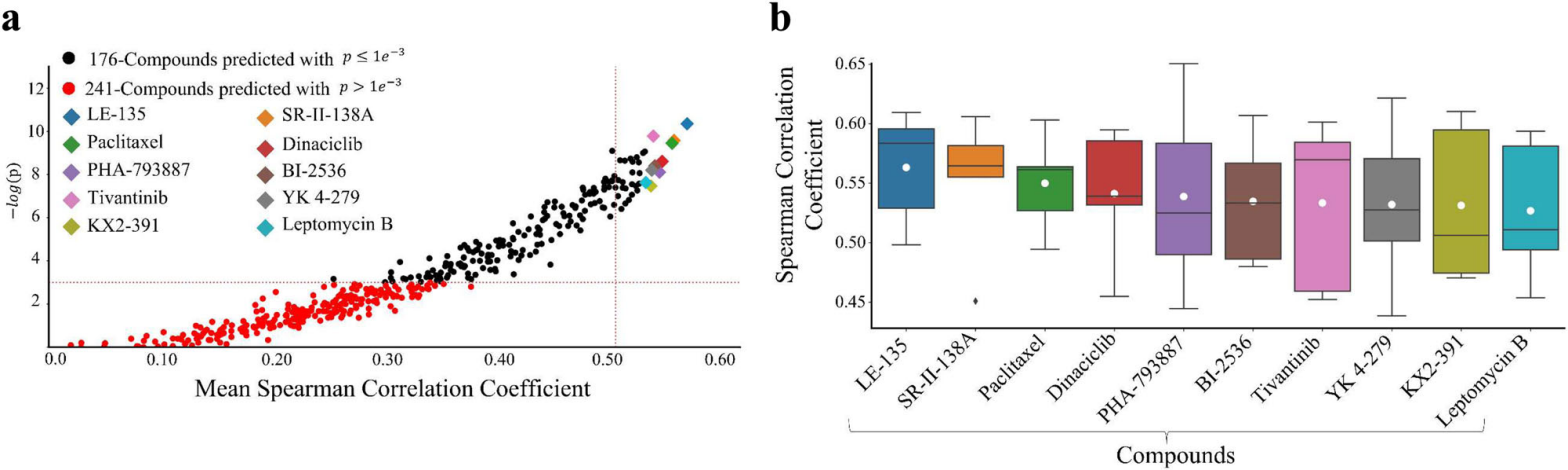
Predictibility of patient tumour sensitivity to different compounds from histology images. **a** Scatter plot showing the correlation between histology image-based predicted sensitivity and gene expression-based imputed drug sensitivity using Spearman correlation coefficient (SCC) as performance metric. The mean SCC across 5-fold cross-validation is shown along *x*-axis with its corresponding -log10 (FDR corrected *p* value) along *y*-axis. Each dot represents a particular drug and its colour represents the statistical significance of the alignment between the predicted score and ground-truth value. The diamonds highlight the top 10 drugs whose histology image-based-predicted sensitivity closely matches with ground-truth values in terms of SCC. **b** Boxplot showing the distribution of SCC across 5-fold cross-validation for the top 10 best-predicted compounds from histology images. Boxes show quartile values while whiskers extend to data points within 1.5× interquartile range. The black horizontal line in each box represents the median of the distribution, while the white dot represents the mean of the distribution.

Our predictive analysis shows that patient sensitivity to several compounds can be predicted from histology images with high Spearman correlation coefficient (SCC) values and significant *p* values, as shown in Fig. 2. It can be observed that for 186 out of 427 drugs, the sensitivities predicted by our model are significantly correlated ($p\ll 0.001$) with the ground-truth sensitivity estimates, and for the top 10 drugs the mean SCC values are above 0.5. Detailed results for all compounds are provided in Supplementary Data 2 and 3. This shows that the responsiveness of the patient’s tumour to many compounds can be inferred from their histological imaging profile. In addition, we have also provided the results of different ablation studies such as restricting the analysis to only tumour regions of WSIs, using domain-specific feature representations, leaving one site out of validation, and using high-quality vs all WSIs in supplementary materials (Supplementary Figs. 1 and 2).

### Association of drug sensitivity with spatially resolved cellular and histopathological phenotypes

**Fig. 3:**
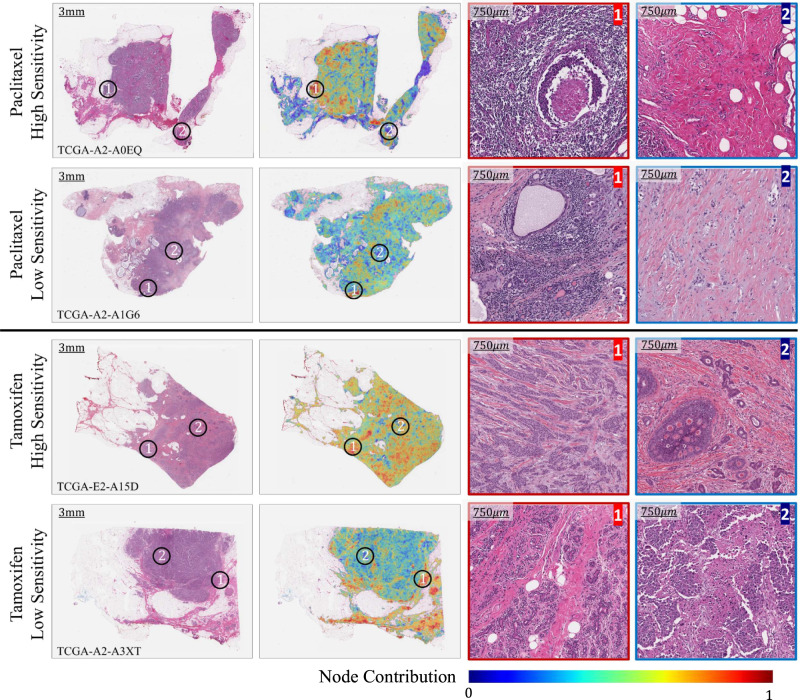
Illustration of different histological patterns within the WSIs associated with patient sensitivity towards Paclitaxel and Tamoxifen. Example WSIs and their corresponding heatmaps are shown for patients being either highly or lowly sensitive to these drugs. The heatmaps use pseudo-colours (blue to red) to highlight the spatially resolved contribution of different regions of the WSI towards the predicted sensitivity. Bluer and redder colour respectively indicate regions of the WSI that contribute the most towards deciding low or high sensitivity. From the WSIs, we extracted magnified versions of regions of interest (ROIs), indicated by the black circles within the WSIs, that are associated with high and low sensitivity of a certain drug. ROIs outlined in red colour are indicative of high sensitivity, while those outlined in red blue colour are indicative of low sensitivity of a certain drug. For an interactive visualisation, please see: tiademos.dcs.warwick.ac.uk/bokeh_app?demo=HiDS.

Pathological assessment of TME in breast cancer plays a pivotal role in predicting tumour behaviour and treatment outcome^38^. The proposed graph-based approach allows the highlighting of spatially localised morphometric patterns associated with the sensitivity of different drugs using node-level prediction score as a guiding signal. Figure 3 shows some example heatmaps highlighting the contribution of different regions of the WSI towards the predicted sensitivity of the patient’s tumour to paclitaxel and tamoxifen. For both drugs, an example WSI and a heatmap are shown for a highly sensitive tumour and a relatively insensitive tumour. The heatmaps show the relative contribution of different regions of the WSI towards the predicted sensitivity estimate of the tumour to a certain drug using pseudo-colours, with dark red colour indicating regions contributing to high sensitivity and dark blue colours corresponding to regions contributing to the prediction of low sensitivity. From the high and low contributing regions, we extracted some sample regions of interest (ROIs) outlined by red and blue colour, respectively, in Fig. 3. The figure illustrates that ROIs associated with high sensitivity to paclitaxel exhibit relatively high proportions of tumour cells as well as lymphocytes. Conversely, ROIs indicative of low sensitivity to paclitaxel are characterised by a notable myxoid change in the stroma.

Regarding tamoxifen, ROIs indicative of high sensitivity are characterised by tumour cells that have relatively low nuclear pleomorphism. Conversely, in ROIs indicative of low sensitivity, the presence of necrosis, increases in mitotic count and cribriform DCIS (ductal carcinoma in situ) can be observed.

### Histological patterns associated with sensitivity to different drugs

**Fig. 4:**
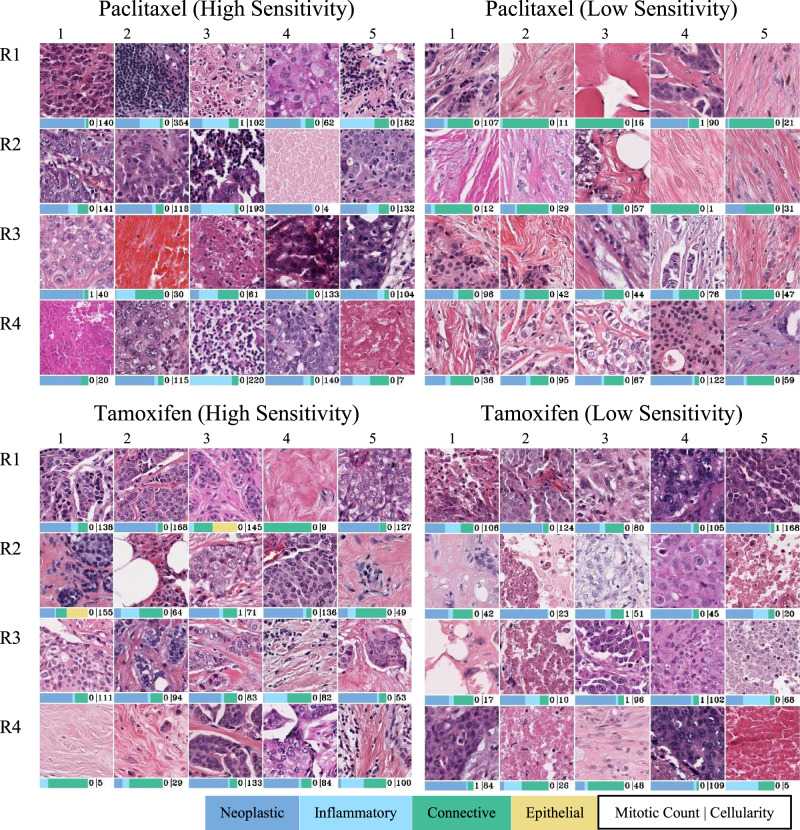
Representative patches (each measuring 128 × 128 μm) of Paclitaxel and Tamoxifen high and low sensitivity. The bars below each patch respectively show its cellular composition in terms of relative counts of four different cell types (neoplastic, inflammatory, connective and epithelial), mitotic counts and overall cellularity (cell counts). Sheet of pleomorphic tumour cells (e.g., R11, R14, R21, R22, R25, R31, R35, R42 and R45) and necrosis (R13, R24, R33 and R41) can be seen in patches relating to paclitaxel high sensitivity. In patches relating to paclitaxel low sensitivity and tamoxifen high sensitivity, the consistent feature is dense sclerotic stroma. In patches associated with tamoxifen low sensitivity, the cells are more pleomorphic (R12, R14, R23, R24 and R33) with some evidence of necrosis (R11, R25, R32, R35 and R42).

We investigated the association of visual histological patterns in the WSIs with the sensitivity of drugs by identifying exemplar patches (of size $512\times 512$ pixels at a spatial resolution of 0.25 microns-per-pixel) for the high and low sensitivity of each drug using clustering. For these patches, we also computed the cellular composition (counts of neoplastic, inflammatory, connective, and epithelial cells), overall cellularity and mitotic counts. These visual patterns or histological motifs can be used as a potential indicator to guide therapeutic decision making. Figure 4 shows representative patches for patients showing high or low sensitivity to paclitaxel and tamoxifen. The most prominent feature in patches representative of high sensitivity to paclitaxel is sheets of pleomorphic tumour cells. In addition, some patches also exhibit evidence of necrosis and lymphocytic infiltration. In contrast, dense sclerotic stroma is the most consistently observed feature across patches representative of low sensitivity to paclitaxel. Regarding tamoxifen, we observed similar histological patterns relating to high sensitivity as the ones present in patches predicting low sensitivity to paclitaxel. For example, both tamoxifen high sensitivity and paclitaxel low sensitivity representative patches are more sclerotic with less pleomorphic tumour cells. However, in patches indicative of patient low sensitivity to tamoxifen the cells are more pleomorphic with some evidence of necrosis.

In addition to paclitaxel and tamoxifen, Supplementary Table 1 lists several other compounds along with their histological patterns of sensitivity and insensitivity. The table also provides information about the gene name of the protein targeted by the compound, compound activity, and compound FDA approval status.

### Association of drug sensitivity with pathologist-assigned histological phenotypes

**Fig. 5:**
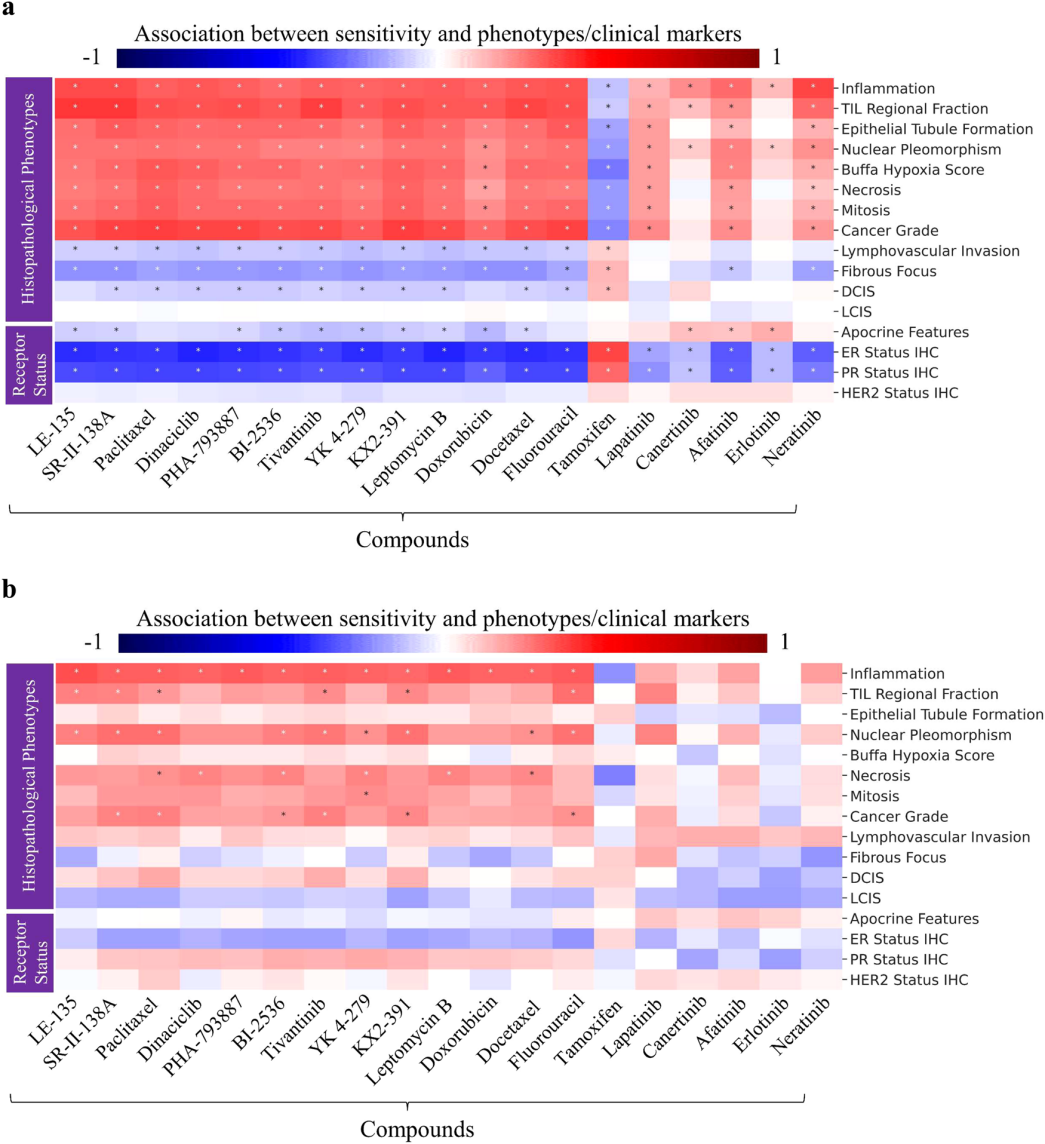
Association of drug sensitivity predicted by our model with pathologist-assigned WSI-level histological phenotypes and breast cancer receptor status. Compounds are shown along *x*-axis, and histological phenotypes and receptor status are shown along *y*-axis. Red and blue colours indicate the degree of association (Kendall’s tau correlation) between the predicted sensitivity of compounds and a specific histopathological phenotype or clinical marker. Bluer colour show strong negative correlation while strong positive correlation is shown using dark red colour. Boxes in the heatmap marked with an asterisk (*) indicate a statistically significant association (determined by the two-sided Wilcoxon rank-sum test, $p\ll 0.05$). Panel (**a**) shows the association for patients with Invasive Ductal Carcinoma (IDC), while panel (**b**) for patients Invasive Lobular Carcinoma (ILC). TIL tumour infiltrating lymphocytes, LCIS lobular carcinoma in situ, DCIS ductal carcinoma in situ, ER estrogen receptor, PR progesterone receptor, HER2 human epidermal growth factor receptor 2.

We validated the predicted sensitivity estimates for several drugs and their associated histological patterns (identified using the proposed pipeline) by calculating Kendall’s tau correlation between image-based predicted sensitivities of drugs and pathologist-assigned WSI-level histological phenotypes. Owing to the difference in morphology^39^ and diagnosis^40^ of invasive ductal carcinoma (IDC) and invasive lobular carcinoma (ILC), the two most common histological subtypes, we analysed the association for each subtype separately. We found IDC patients predicted sensitivities to chemotherapy drugs (e.g., paclitaxel, docetaxel, doxorubicin, etc.) positively associated (FDR corrected Wilcoxon rank-sum test $p\ll 0.05$) with cancer grade, mitosis, inflammation, necrosis, nuclear pleomorphism, epithelial tubule formation, TIL regional fraction^41^ and buffa hypoxia score (see Fig. 5a). However, an opposite association was observed for hormonal therapy drug tamoxifen. For example, tamoxifen is likely to be more effective for patients with low-grade cancer and reduced hypoxic tumour features, while paclitaxel is likely to be more effective for patients with aggressive cancer characteristics.

Regarding ILC patients, the degree of association between patients’ sensitivity to compounds and pathologist-assigned phenotypes (Inflammation, TIL regional fraction, nuclear pleomorphism, necrosis, and cancer grade) is weaker compared to IDC patients (see Fig. 5b). For example, ILC patients’ sensitivity to chemotherapy compounds (e.g., SR-II-138A, paclitaxel, tivantinib, KX2-391 and Fluorouracil) show relatively weak association with cancer grade compared to IDC patients. In addition, ILC patients’ image-based predicted sensitivity to chemotherapy drugs shows negligible association with epithelial tubule formation (see Fig. 5b).

For both these subtypes, we also analysed the association between patient’s gene expression-based imputed sensitivity to compounds and histological phenotypes, and we observed roughly similar association patterns (see Supplementary Fig. 3) to those seen when using image-based predicted sensitivity.

### Association of drug sensitivity with Receptor status

We analysed the association of image-based predicted sensitivity with routine breast cancer clinical markers (ER, PR and HER2 status) with the end goal of explaining the model-predicted sensitivity in terms of these markers. As expected, we found that patients’ sensitivity to compounds can be explained in terms of these markers. For example, for IDC patients, we found their image-based predicted sensitivities to most of chemotherapy drugs (e.g., paclitaxel, docetaxel, etc.) to be negatively associated with ER and PR status (see Fig. 5a). However, we found a positive association between patients’ sensitivity to tamoxifen (a hormone therapy drug) and their ER/PR status. These results are in line with previous studies that have reported the effectiveness of tamoxifen in low-grade ER-positive patients treated with tamoxifen^42,43^. Regarding ILC patients, we see a small degree of positive correlation between ER positivity and tamoxifen sensitivity, while for chemotherapy drugs, we see a negative correlation, as can be seen in Fig. 5b. Finally, for both IDC and ILC patients, our analysis does not show significant association (FDR corrected Wilcoxon rank-sum test, $p\gg 0.05$) between patients’ image-based predicted sensitivity to compounds and their HER2 status (see Fig. 5b).

### Correlation of drug sensitivity with cellular composition

**Fig. 6:**
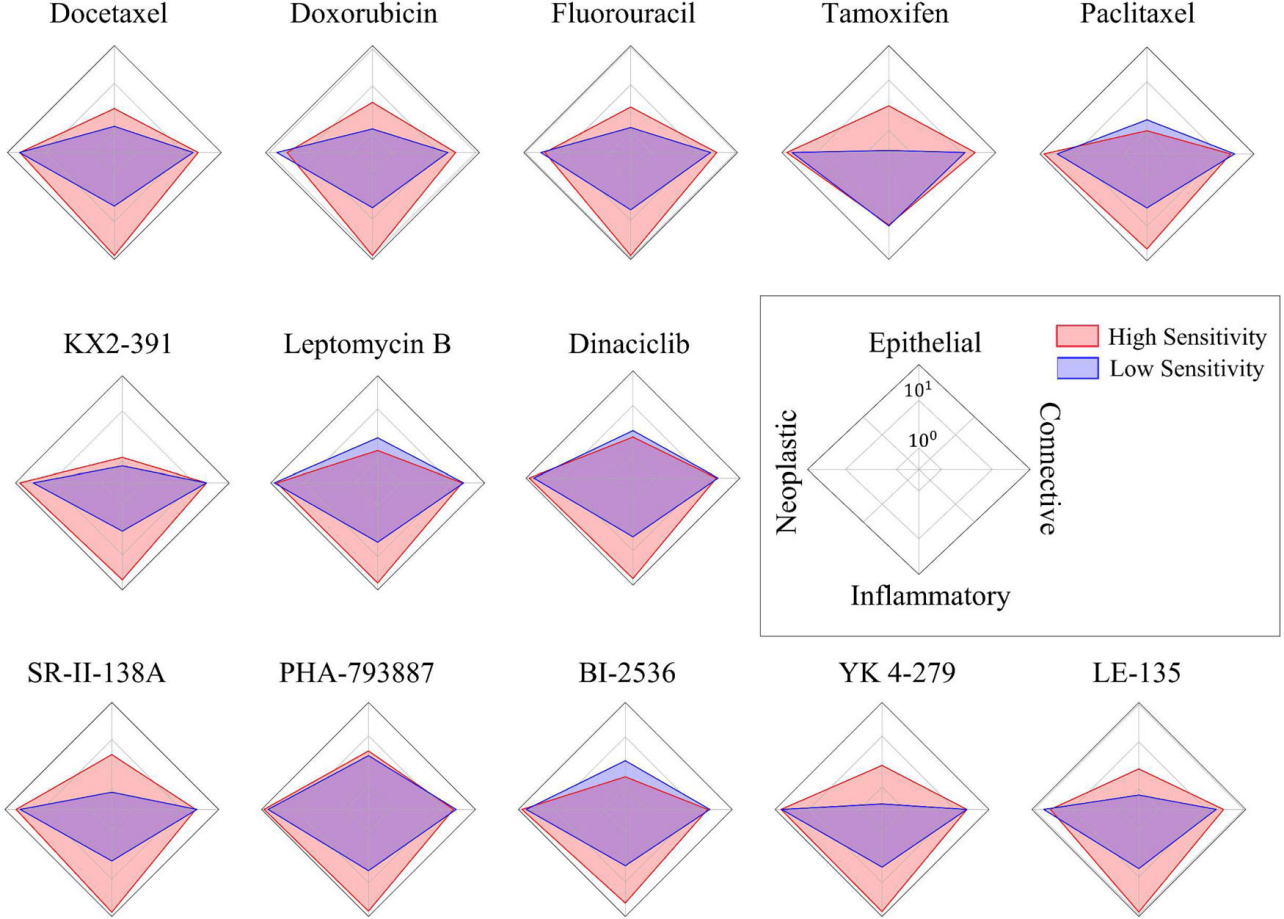
Radar plots of drug sensitivity and cellular composition. The plots show the association of relative counts of different type of cells with high and low sensitivity to a certain drug. Each axis of the plot represents a particular cell type, and the length of the axis shows their counts on a log scale. For example, the radar plot of Paclitaxel shows that patients who are highly sensitive to Paclitaxel have relatively higher numbers of inflammatory and neoplastic cells compared to those who are less sensitive.

We analysed the relative proportion of neoplastic, inflammatory, connective and epithelial cells in WSI patches contributing to high and low sensitivity, as shown by a radar plot of the various cellular counts in Fig. 6. For all drugs other than tamoxifen, image patches representative of high sensitivity have a relatively higher proportion of inflammatory cells compared to patches associated with low sensitivity. Apart from inflammatory cells, the sensitivity of different compounds shows an association with different patterns of cellular composition. For example, paclitaxel and KX2-391 show high sensitivity when the counts of neoplastic cells are relatively higher. As for the remaining drugs, the counts of neoplastic cells do not show notable differences in high- and low-sensitive groups. Similarly, BI-2536, Dinaciclib, Paclitaxel, and Leptomycin B show high sensitivity when the count of normal epithelial cells is relatively low, while the remaining drugs show high sensitivity when epithelial cells are higher in number. Among the listed drugs, a different pattern is shown by tamoxifen, which is highly sensitive when the normal epithelial cell count is relatively high, whereas its sensitivity is low in tumour-rich regions (i.e., patches with relatively high neoplastic cell counts with almost no normal epithelial cells). Finally, for all compounds (except Dinaciclib, Tivantinib, and Leptomycin B), high-scoring patches of sensitivity/insensitivity show significant (FDR corrected *p* ≪ 0.05) differences in patch-level connective cell counts (see Supplementary Fig. 4).

### Correlation between drug sensitivity and inflammatory to neoplastic cell ratio

**Fig. 7:**
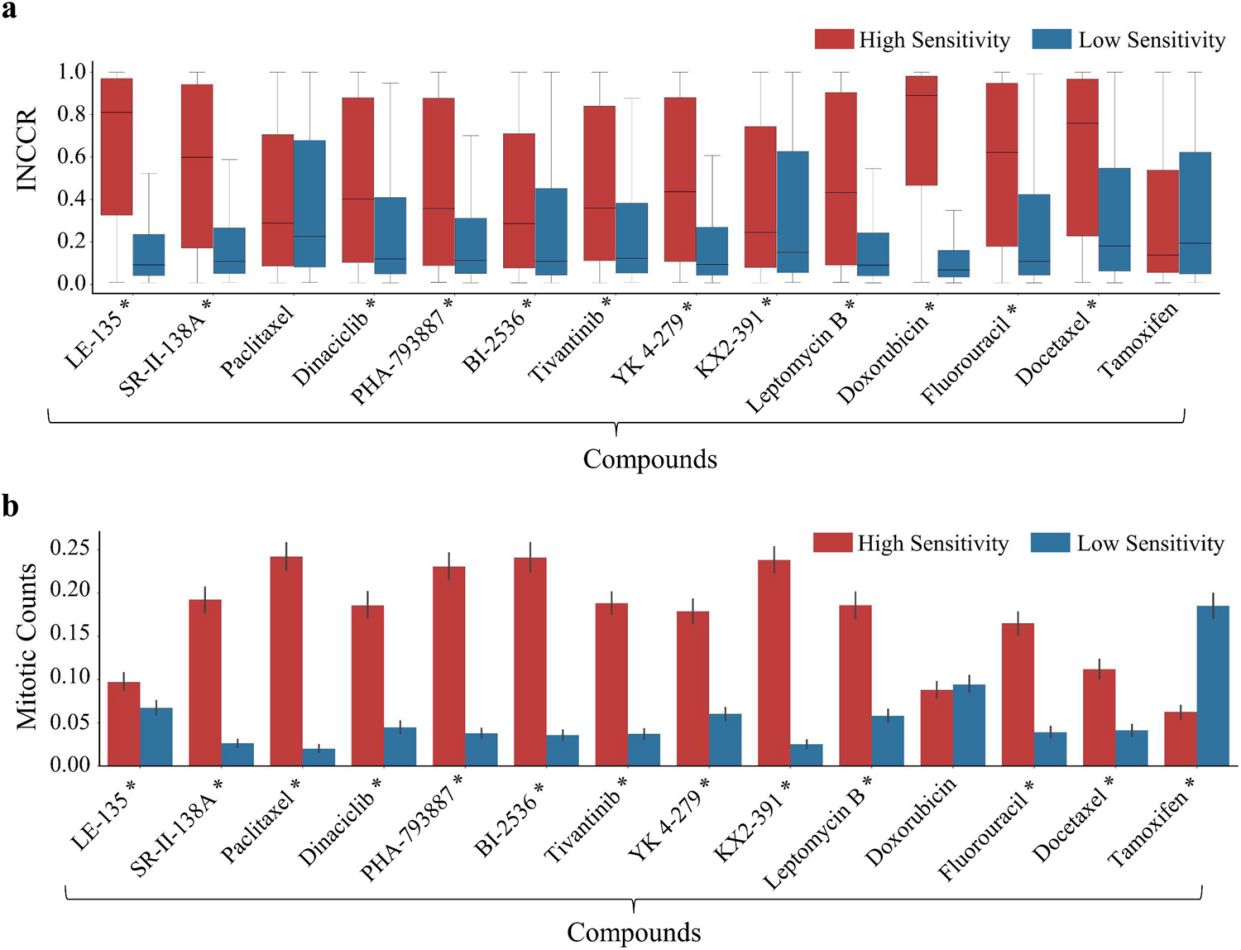
Association of drugs sensitivity with neoplastic to inflammatory cell count ratio (INCCR) and mitotic counts. Plots showing the association of compound sensitivity with **a** inflammatory to neoplastic cell counts ratio (INCCR) and **b** mitotic counts. Compounds are shown along *x*-axis, while the distribution of INCCR/Mitotic count is shown long *y*-axis. Red and blue colours represent the high- and low-sensitive groups, respectively. Compounds with * next to their name show statistically significant differences (two-sided Wilcoxon rank-sum test, $p\ll 0.05$) in INCCR (in case of **a**) and mitotic counts (in case of **b**) between patches indicative of high and low sensitivity.

We assessed the association between patch-level inflammatory to neoplastic cell count ratio (INCCR) and sensitivities of several drugs. We found that most chemotherapy drugs show high sensitivity when the patch-level INCCR is higher, whereas the opposite is true for tamoxifen, a hormonal therapy drug (see Fig. 7a). We validated our findings statistically using Wilcoxon rank-sum test. Notably, for most compounds (except paclitaxel and tamoxifen), patches associated with high sensitivity exhibit significantly ($p\ll 0.05)$ higher INCCR compared to patches associated with low sensitivity.

### Correlation between drug sensitivity and patch-level mitotic counts

We examined how patch-level mitotic counts are related to the sensitivity of various drugs. Our analysis shows that most chemotherapy drugs tend to be more sensitive when the patch-level mitotic counts are higher, whereas the opposite is true for tamoxifen (a hormonal therapy drug), which shows high sensitivity when patch-level mitotic counts are lower, as can be seen in Fig. 7b. For example, patients’ shows high sensitivity to paclitaxel when patch-level mitotic counts are relatively higher. We validated these findings statistically using Wilcoxon rank-sum test. Apart from doxorubicin, all drugs listed in the figure show significant ($p\ll 0.05)$ difference in patch-level mitotic counts between patches associated with high and low sensitivity.

We also analysed the association of compound sensitivity with patch-level interaction of mitotic counts and INCCR using a regression model (see Methods section ‘Cellular composition and statistical analysis’). For most compounds (except tamoxifen and LE-135), we found that patch-level mitotic counts and INCCR have a joint multiplicative effect on drug sensitivity (FDR corrected $p\ll 0.05$) (see Supplementary Table 2) over and above their direct effects on sensitivity.

## Discussion

In this study, we proposed a deep learning pipeline to predict the sensitivity of breast cancer tumours to various drugs from WSIs. To explicitly model the local and global histological patterns in WSI, we employed our recently proposed ${{SlideGraph}}^{\infty }$ pipeline^37^, which constructs a graph representation of the WSI first, and then train a GNN that predict WSI-level sensitivity while also highlighting the spatially resolved contribution of different regions within the WSI. We analysed the performance of ${{SlideGraph}}^{\infty }$ in predicting the sensitivity of TCGA breast cancer patients to 427 drugs using only routine H&E histology images. Almost one-half (186 out of 427 drugs) showed a statistically significant correlation ($p\ll 0.05)$ between the patients’ predicted sensitivity to the drugs based on histology images and their imputed sensitivity based on gene expression. Moreover, we also identified histological patterns associated with high and low sensitivities to several drugs in terms of cellular composition, mitotic counts and histological motifs.

The proposed approach is fundamentally different from other approaches that predict patient sensitivity to different drugs by associating genomic and pharma data from cancer cell lines using ML^14,16^. Most of these methods aim to discover patterns of gene expression that play a role in determining patient responsiveness to a particular drug. To the best of our knowledge, this is the first study to associate the sensitivity of anti-cancer drugs with tissue phenotypic information from routine histology images. We anticipate that direct association of drug sensitivity with histological profiles will help in discovering new histological patterns that can be analysed by a pathologist to assist with therapeutic decision making for individual patients. The ground-truth sensitivity estimates used for training our histology-based model were obtained using a model trained on pharmacogenomic data of cancer cell lines. The motivation behind using cancer cell line for ground-truth sensitivity estimates is that it allows virtual screening of patients for a large number of compounds in a relatively short time and with minimal cost. We analysed the effectiveness of the proposed method on breast cancer data only, but it can be extended to other cancer types, subtype or patient populations examined by the investigator. Finally, the proposed pipeline allows virtual screening of cellular response of biological specimens to lead compounds studied during drug discovery.

The employed image-based sensitivity prediction approach allows mapping patient WSI-level drug sensitivity to localised cellular and histological phenotypes using model node-level prediction scores. An illustration of this can be seen in Fig. 3. For example, for paclitaxel-sensitive tumours, the model has assigned high score to regions of WSI with relatively high proportions of tumour cells as well as lymphocytes, which aligns with previous research demonstrating that tumour infiltrating lymphocytes (TILs) can act as an independent predictor of a patient tumour’s sensitivity to chemotherapy drugs^44^. In contrast, for paclitaxel insensitive tumours the model has assigned high score to regions of WSI that exhibit a significant myxoid stromal alteration, a characteristic previously found associated with unfavourable overall survival (OS) and relapse-free survival (RFS) in triple-negative breast cancer^45^. Similarly, for tamoxifen, a hormonal therapy drug, the model has associated regions of the tumour characterised by low nuclear pleomorphism to sensitivity, whereas areas of tumour with the presence of necrosis and increased mitotic activity to insensitivity. This is in line with previous studies that have found a correlation of necrosis with larger tumour size and higher cancer grade, and hence it may be postulated that highly necrotic regions may contribute to relatively low sensitivity to tamoxifen^46^. This analysis shows that the proposed approach has associated relevant histological features in the WSIs with the sensitivity and insensitivity of drugs.

Despite the promising results, the proposed approach has several limitations. Similar to other studies aimed at analysing response to drugs^19,47,48^, the ground-truth sensitivity estimates used by our method are obtained based on gene expression data. While other studies^48,49^ have shown that drug sensitivity estimates based on gene expression profile are accurate and useful, extensive validation is still needed as gene expression may not be the only factor responsible for drug sensitivity. For example, epigenetic factors and proteomic expression changes can impact drug sensitivity without having any direct gene expression change. Another fundamental limitation stems from the use of ground-truth sensitivity estimates inferred from cancer cell lines which, while being low cost and high throughput, lack the microenvironment components that are known to influence response to therapy^50^.

Besides this, while we have demonstrated that the proposed approach can accurately predict patients’ sensitivity to several compounds from WSIs, it is important to highlight that the model is still not able to predict the sensitivity of all compound classes. For example, the model predictions for HER2-inhibitors such as Lapatinib, Erlotinib, Afatinib, Canertinib, and Neratinib consistently show low median Spearman correlation values of 0.20, 0.18, 0.33, 0.31, and 0.29, respectively. This could potentially be attributed to the poor predictability of HER2 status from WSIs of H&E-stained tissue section shown by the previous study^35^. This limitation could also explain the weak association between image-based predicted sensitivity of compounds and HER2 status (Fig. 5), even though gene expression-based imputed sensitivities of compounds show an association with HER2 status (Supplementary Fig. 3).

Deep learning has been proven valuable in predicting cancer subtypes^23,24^, patient survival^26^, mitosis detection^27^, DNA methylation patterns^28^, cellular composition^29^, and tumour mutation burden^31^ from WSIs of H&E-stained tissue sections. Taking a step forward, this proof of principle study has demonstrated that deep learning can predict patients’ sensitivity to a number of compounds from routine histology images. We validated the proposed approach extensively through site-independent validation (Supplementary Fig. 1d), associating our model-predicted sensitivities with pathologist-assigned histological phenotypes and IHC-evaluated receptor status, and hypoxia scores. However, the proposed approach still needs a more stringent validation on a large multi-centric independent cohort from a randomised control trial (RCT) before going to clinical implementation.

## Methods

### Ethics statement

All samples used in the study were obtained with research consent and ethics approvals as indicated in the consent and ethics statements for the Cancer Genome Atlas (TCGA)^51,52^, CCLE^9^ and CTRP^11^.

### Acquisition of whole slide images and drug-response data

We collected 1133 WSIs of Formalin-Fixed Paraffin-Embedded (FFPE) H&E-stained tissue section of 1084 breast cancer patients from the Cancer Genome Atlas (TCGA)^51,52^. The gene expression profile-based drug sensitivity estimates of 936 TCGA breast cancer patients for 427 compounds were obtained from the work of Gruener et al.^15^. To limit the impact of various artefacts, we excluded WSIs that met any of the following criteria: (1) containing extensive blurry areas; (2) having abnormal staining with minimal informative tissue regions; or (3) lacking baseline resolution information. After filtering, in total we used WSIs of 551 patients along with their imputed sensitivity to 427 drugs in our analyses.

### Pre-processing

For each WSI, we first identify the viable tissue areas and exclude regions with artefacts (such as pen-marking or tissue folding) by applying a U-Net-based segmentation model from the TIAToolbox^53^. The output tissue mask from the model highlights viable tissue regions with non-zero-valued pixels, whereas background and regions with artefacts are represented by zero-valued pixels. Based on these tissue masks, we extract patches of size $512\times 512$ pixels at a spatial resolution of 0.25 microns-per-pixel (MPP) from each WSI. We only keep patches (both tumour and benign) that have more than 40% of viable tissue in terms of proportion of pixels in a patch. We analysed both tumour and non-tumour patches based on the hypothesis that patients sensitivity to compounds can be influenced by interactions among different cell types (including stromal cells) in the TME^54–56^.

The target drug sensitivity data are converted into z-score prior to prediction with high sensitivity corresponding to lower AUC-DRC for that compound and vice versa.

### Graph modelling of whole slide image

A graph is defined by a set of nodes or vertices $V$, and an edge set $E$. In the context of this application, the set $V=\left\{{{\boldsymbol{v}}}_{{\boldsymbol{i}}}|i=1,\ldots N\right\}$ consists of set of patches contained in the WSIs. Each node ${{\boldsymbol{v}}}_{{\boldsymbol{i}}}=\left({{\boldsymbol{g}}}_{{\boldsymbol{i}}}{\boldsymbol{,}}\,{{\boldsymbol{h}}}_{{\boldsymbol{i}}}\right)$ encodes the spatial location (${{\boldsymbol{g}}}_{{\boldsymbol{i}}}$) and feature representation (${{\boldsymbol{h}}}_{{\boldsymbol{i}}}$) of a patch, where ${{\boldsymbol{h}}}_{{\boldsymbol{i}}}\,{{\in }}\,{{\mathcal{R}}}^{{\boldsymbol{1024}}}$ is 1024-dimensional feature representation of a patch extracted from ShuffleNet^57^ pretrained on ImageNet^58^. The edge set $E$ is obtained by linking each node with their neighbouring nodes (within 4000 pixels) using Delaunay triangulation. An edge ${e}_{{ij}}\in E$ exists in the set $E$ if two nodes ${v}_{i}$ and ${v}_{j}$ are connected.

### Prediction of drug sensitivity using Graph Neural Network (GNN)

We utilise a GNN to predict node-level and WSI-level sensitivity of patients to a set of drugs $D$ from their corresponding WSI-graphs. More specifically, we used ${{SlideGraph}}^{\infty }$ with slightly modified architecture^37^. The multi-output GNN model predicts patch-level and WSI-level sensitivity of patients to $D$ different drugs in an end-to-end manner. Node-level representation (feature embedding of a patch) undergoes a series of EdgeConv layers $L=\left\{\mathrm{1,2,3}\right\}$. In each EdgeConv layer^59^, the representation of each node in the graph is updated by gathering information from its neighbouring nodes using message passing. This aggregation process generates embeddings that are utilised in the subsequent layers. The output embedding of an EdgeConv for a node at index $m$ in layer $l$ can be mathematically written as follows:

$${{\boldsymbol{h}}}_{m}^{l}=\sum _{k\in {\mathcal{N}}\left(m\right)}{{\mathcal{H}}}^{l}\left({{\boldsymbol{h}}}_{m}^{l-1}\parallel {{\boldsymbol{h}}}_{k}^{l-1}-{{\boldsymbol{h}}}_{m}^{l-1}\right)$$

In the above equation, the term ${{\boldsymbol{h}}}_{m}^{0}$ is the initial embedding of node $m$ which is equivalent to ${{\boldsymbol{h}}}_{m}$. The symbol ${\mathcal{N}}\left(m\right)$ represents the neighbouring nodes of $m$ and ${{\mathcal{H}}}^{l}$ denotes a multi-layer perceptron at layer $l$. The EdgeConv operation updates the feature representation of a node ${{\boldsymbol{h}}}_{m}^{l}$ by aggregating information from its neighbouring nodes ${\mathcal{N}}\left(m\right)$. In the case of $\left|L\right|=3$, each node is expected to gather information from the neighbouring nodes that are 4-hops apart in the WSI-level graph.

The node-level embedding ${{\boldsymbol{h}}}_{m}^{l}$ of a node ${{\boldsymbol{v}}}_{m}=\left({{\boldsymbol{g}}}_{{\boldsymbol{j}}},{{\boldsymbol{h}}}_{{\boldsymbol{j}}}\right)\in V$ is passed as input to a multi-layer perceptron ${f}_{l}\left({{\boldsymbol{v}}}_{m}\right)=f\left({{\boldsymbol{h}}}_{m}^{l}\right)$ for generating node-level prediction score. The patch-level prediction score of patient sensitivity to different drugs can be obtained by aggregating node-level prediction score across all layers $f\left({{\boldsymbol{v}}}_{m}\right)=\mathop{\sum }\nolimits_{l=0}^{L}{f}_{l}\left({{\boldsymbol{v}}}_{m}\right)$. WSI-level prediction score $F(G)$ is then obtained by pooling and aggregating node-level prediction score $F\left(G\right)={\sum }_{\forall m\in V}f\left({{\boldsymbol{v}}}_{m}\right)$.

The trainable parameters of both EdgeConv layers and node-level regressor are learned in an end-to-end manner using backpropagation. During training, for a batch of size $N=16$ patients, the predicted sensitivity values for $d=\{1\ldots D\}$ drugs are compared with ground-truth values using pairwise ranking loss, mathematically formulated as follows:

$${\mathcal{L}}=\sum _{d}\sum _{(a,b)\in {P}_{d}}\max \left(0,1-\left({F}^{d}\left({G}_{a}\right)-{F}^{d}\left({G}_{b}\right)\right)\right)$$

In the above formulation, ${P}_{d}=\left\{\left(a,b\right)|{y}_{a}^{d}\, > \,{y}_{b}^{d},{a},b=1\ldots .N\right\}$ represents all pairs (*a, b*) where the ground-truth sensitivity estimate of patient $a$ is greater than patient $b$ for drug $d$. Minimising the loss function will enforce the model to rank highly sensitive patients higher than the low-sensitive ones for all drugs.

### Model training and evaluation

We trained and evaluated the performance of the proposed method using 5-fold cross-validation in which the data were partitioned into non-overlapping 80/20 training and test splits. For validation, we randomly selected 20% data from the training set and use it for parameter tuning and optimisation. We trained the model for 300 epochs using adaptive momentum-based optimiser^60^ with a learning rate of 0.001 and weight decay of 0.0001 on the training set using a batch size of 16. We stopped the model training if the validation loss was not minimising over 20 consecutive epochs. During training, we used a queue of size 10 and put the best model in the queue based on its performance over the validation set. For the test set, we aggregated the scores of the 10 best models from the queue to obtain a final prediction score. To evaluate the model performance for a given drug on the test set, we computed SCC value between the ground-truth and predicted sensitivity values with its associated *p* value. For a given drug, the *p* value associated with SSC values across multiple cross-validation runs was combined by calculating twice the median *p* value (p50) as a conservative estimate for statistical significance^61^. For predictive performance evaluation, we used the *p* value and mean SCC as performance metrics.

### Identification of histological patterns associated with drug sensitivity

To identify histological patterns associated with high and low sensitivity of a certain drug, we divided patients into two classes (high sensitivity and low sensitivity). For each class, we selected top 50 patients based on absolute difference between imputed sensitivities and model-predicted sensitivities. From the WSIs of patients belonging to highly sensitive groups we extracted the highest-scoring (based on node-level score) 1% patches, while for low-sensitive cases, we extracted the lowest-scoring 1% patches. Within each class, we then clustered the patches to uncover visual patterns associated with high and low sensitivity using 25-medoid clustering^62^. After clustering, we obtained 25 visual patterns representative of high and low sensitivity of a certain drug.

### Cellular composition and statistical analysis

We analysed the cellular composition of high and low-scoring patches in their respective high- and low-sensitive group using our in-house state-of-the-art cellular composition predictor ALBRT^29^. For a given patch, ALBRT generated a four-dimensional vector representing the counts of neoplastic, inflammatory, connective, and epithelial cells present in a patch. ALBRT was originally trained on patches of size $256\times 256$ pixels at a spatial resolution of 0.25 MPP, so we tiled each patch of size $512\times 512$ pixels into four subpatches and aggregated the ALBRT predicted cellular composition. The patch-level inflammatory to neoplastic cell ratio was computed based on the cellular composition. This was done by dividing the count of inflammatory cells by the sum of neoplastic and inflammatory cell counts.

### Estimation of mitotic counts

The mitosis detection was done using the state-of-the-art mitosis detection method called MDFS (mitosis detection: fast and slow)^63^. The MDFS method follows a two-stage approach to detect mitotic candidates. It first detects the mitotic candidates using a convolutional neural network (CNN) and then subsequently refines the prediction by training a CNN classifier. For more details, interested user is referred to MIDOG challenge paper^27^. After detecting the mitotic figures, we estimated the patch-level mitotic counts by counting all the detected mitoses in the patch.

### Multivariate regression analysis of mitotic counts/INCCR and compounds sensitivity

We analysed the combined effect of patch-level interaction of mitotic counts and INCCR on compounds sensitivity using a logistic regression model. Specifically, for each compound we extracted the top 1% highest-scoring patches (based on node-level scores) from WSIs of highly sensitive cases, and the bottom 1% lowest-scoring patches from WSIs of low-sensitivity cases. We then employed a logistic regression model that uses patch-level mitotic counts, INCCR, and their interaction (INCCR × mitotic count) as features, and the class label of patch as target (i.e., 1 for highly scoring patches of sensitivity, while 0 for insensitivity). For each compound, the combined effect of mitotic counts and INCCR on sensitivity was subsequently deduced from the regression coefficients learned by the model.

### Predicting drug sensitivity exclusively based on tumour regions within WSIs

We assessed the predictability of drugs sensitivity using only tumour tiles of the WSIs as an ablation experiment. The tumour tiles were selected using ALBRT patch-level neoplastic-epithelial cell proportion as proxy. Specifically, a patch is labelled as a tumour patch if the proportion of neoplastic cells within a patch is above 5%. Following this criterion, we stratified the tissue content into tumour and non-tumour regions, and subsequently analysed the predictive performance of ${{SlideGraph}}^{\infty }$ using the same experimental setup and evaluation protocol as employed for the model trained on both tumour and non-tumour patches.

### Predicting drug sensitivity using latent representation from model pretrained on histology images

Self-supervised learning (SSL) is emerging as a promising method for extracting robust representation from WSI. A recent study^64^ has shown that using latent representation from a model trained on histology images in a self-supervised manner can predict the mutation status of a number of genes with higher accuracy compared to using representation from a model pre-train on natural images (i.e., ImageNet). Inspired from this, as an ablation study, we assessed the predictability of sensitivity of different drugs using latent representation from RetCCL, a model trained on histology images^65^. As RetCCL was originally trained on patches of size $1024\times 1024$ pixels at a spatial resolution of 0.50 MPP, therefore, for obtaining patch-level embedding we extracted the WSI patches at the same dimensions and magnification. We then used same experimental setup and evaluation protocol, as employed for ShuffleNet representation^57,58^.

### Analysis of batch effects

A reason for batch effects that are introduced in the WSIs is domain shift. As WSIs in the TCGA-BRCA cohort are from different source site, and each site is likely to use different staining protocol and scanner, resulting in different staining characteristics which can be exploited by the deep learning model. A common approach to remove the stain variability is stain normalization. However, in our recent study, we have shown that the majority of stain normalization methods fail to remove site-specific signature^66^. Consequently, to determine the effect of variations in WSIs across sites on the predictive accuracy of the model, as an ablation study, we trained and evaluated the model performance using leave one site out (LOSO) cross-validation. Specifically, we selected the top 8 sites with at least 5% samples in the dataset belonging to that site. We then assessed the model performance by training the model on data of all sites and left one site out for testing. As a performance metric, for each drug, we reported the mean Spearman correlation across all testing sites.

### Analysing the predictability of sensitivity of drugs sensitivity without filtering WSIs

Apart from analysing the predictability of compounds’ sensitivity using high-quality WSIs from the previous study^67^, as an ablation study, we also analysed the model performance on the whole TCGA-BRCA cohort of 936 patients. We hypothesise that if the model is robust, it would still be able to map relevant histological patterns in patients’ WSIs and their sensitivity to compounds, potentially avoiding a significant drop in model predictive performance.

### Reporting summary

Further information on research design is available in the Nature Research Reporting Summary linked to this article.

### Supplementary information

Supplementary Materials

Supplementary Data 1

Supplementary Data 2

Supplementary Data 3

Reporting summary

## Data Availability

Whole slide images (WSIs) of all TCGA-BRCA patients used in the study can be downloaded from the NIH Genomic Data Common Portal at this link: https://portal.gdc.cancer.gov/ with manifest details included in supplementary materials.

## Code availability

The deep learning pipeline ${{SlideGraph}}^{\infty }$ was developed using PyTorch Geometric and TIAToolbox^53^. Source code and documentation of all Python scripts used in the study can be found at: https://github.com/engrodawood/HiDS.
