# Supplementary material for: Cancer drug sensitivity prediction from routine histology images
Source: NPJ Precis Oncol. 2024 Jan 6;8:5. doi: 10.1038/s41698-023-00491-9 (PMC10771481; doi:10.1038/s41698-023-00491-9)
Supplement: Supplementary file 1 — Supplementary Materials [file 41698_2023_491_MOESM1_ESM.pdf]

Supplemental Materials: Cancer drug sensitivity prediction from routine histology images

Muhammad Dawood, Quoc Dang Vu, Lawrence S. Young, Kim Branson, Louise Jones, Nasir Rajpoot, Fayyaz ul Amir Afsar Minhas

Supplementary Figures

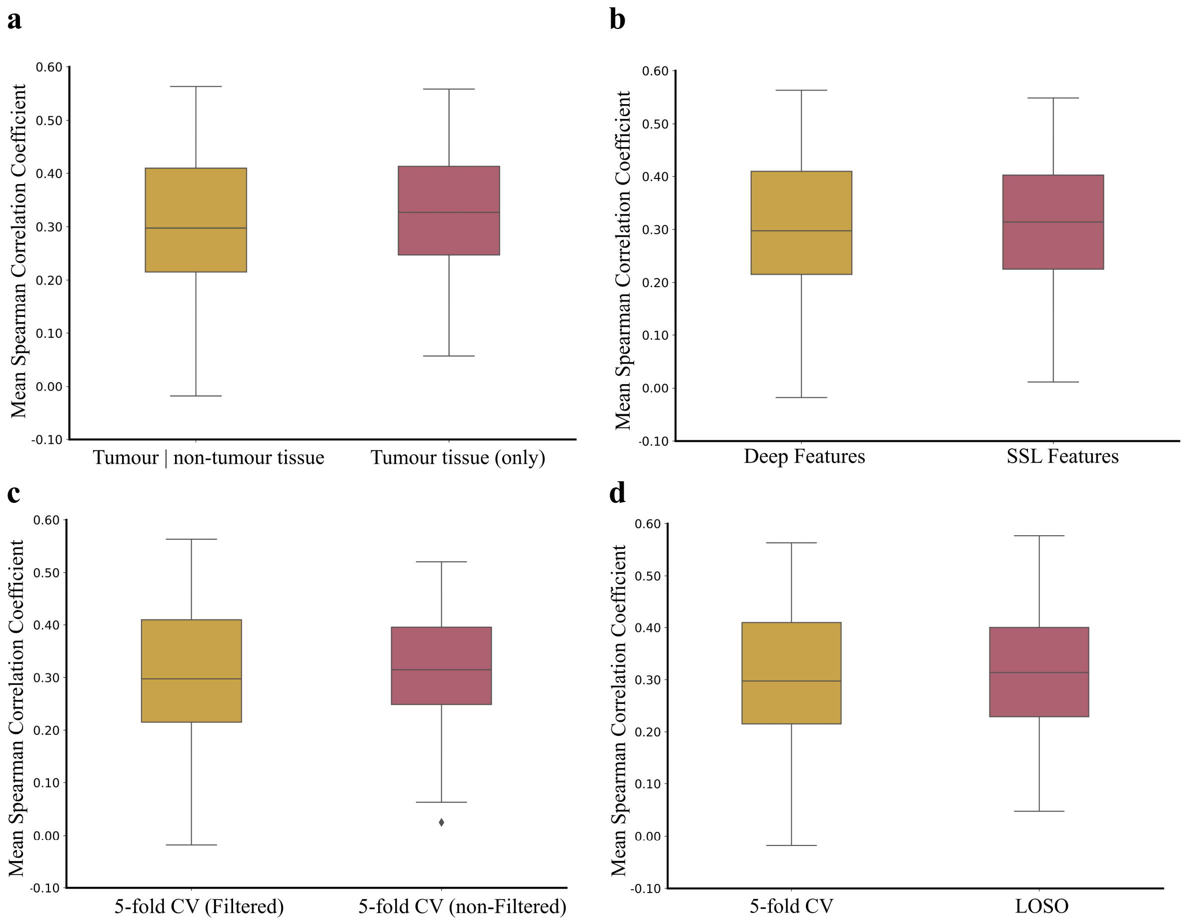

Supplementary Figure 1: Boxplots showing the distribution of mean spearman correlation coefficient (SCC) using different features and model evaluation protocol. a. Impact of slide content stratification on model predictive performance. The plot to the left shows distribution of mean SCC without stratifying tissue content into tumour/benign while the right plot show results when using only tumour tiles. **b.** Influence of patch-level representation on model predictive performance in terms of mean SCC. Left plot shows the distribution of mean SCC when model trained on natural images is used as patch-level encoder, while the plot for when using model trained histology images as patch-level encoder. **c.** Predictability of sensitivity of drugs when using filtered set of whole slide images (excluding WSIs with abnormal tissue staining or low percentage of informative tissue), and without filtering, i.e., using all the TCGA-BRCA patients for which WSIs and imputed drugs sensitivity is available. The plot to the left show the model predictive performance in terms of mean SCC across five cross validation runs, while the plot to the right show the model predictive performance on the non-filtered set. **d.** Comparison of model predictive performance when using different evaluation criteria. The plot to the left shows the distribution of mean SCC across five cross validation runs, while the plot to the right show the results of leave one site out cross validation (LOSO).

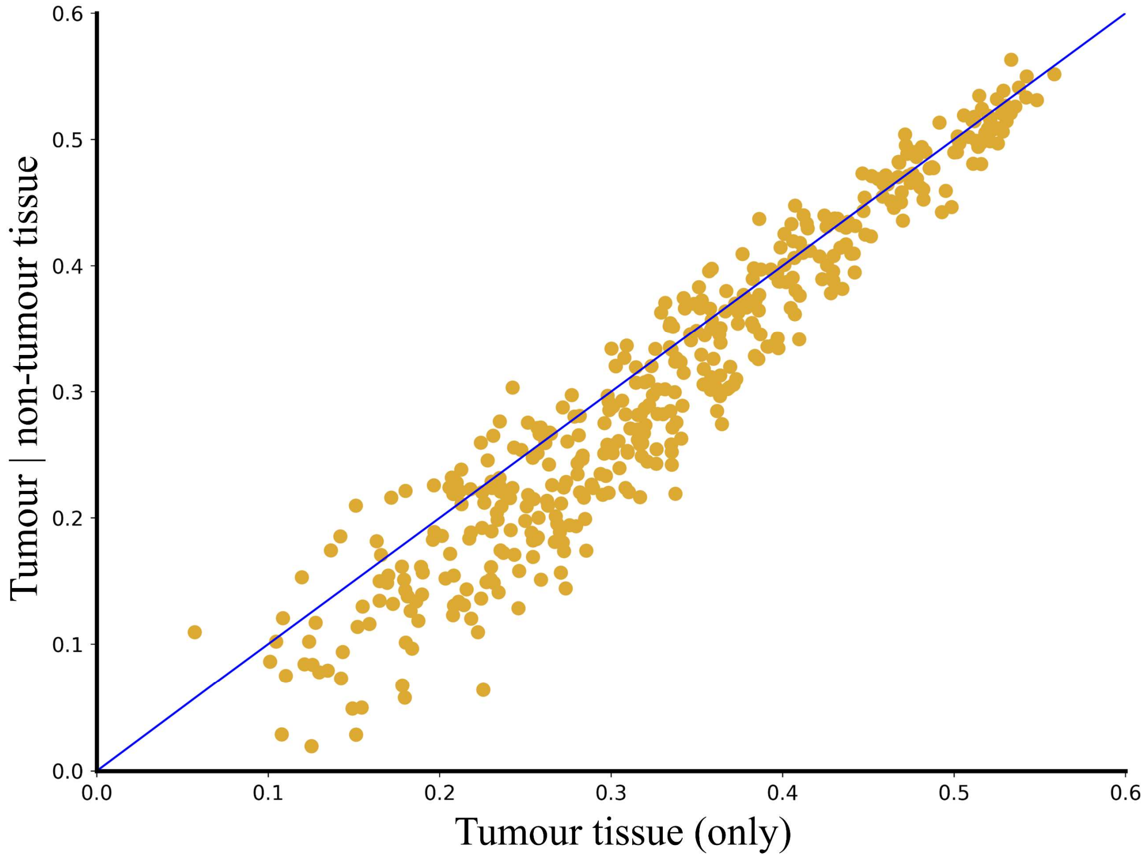

Supplementary Figure 2: Impact of Whole Slide Image content stratification on predictive model performance. The scatter plot compares the predictive performance of two models for predicting patient sensitivity to compounds. One model uses only tumour tissue for inference (x-axis), while the other uses both tumour and normal tissue (y-axis). Each dot represents a particular compound. The median Spearman correlation coefficient at which the sensitivity of a compound is predicted by each model is shown by the x-y position of the dot. The middle blue line show the case of perfect correlation.

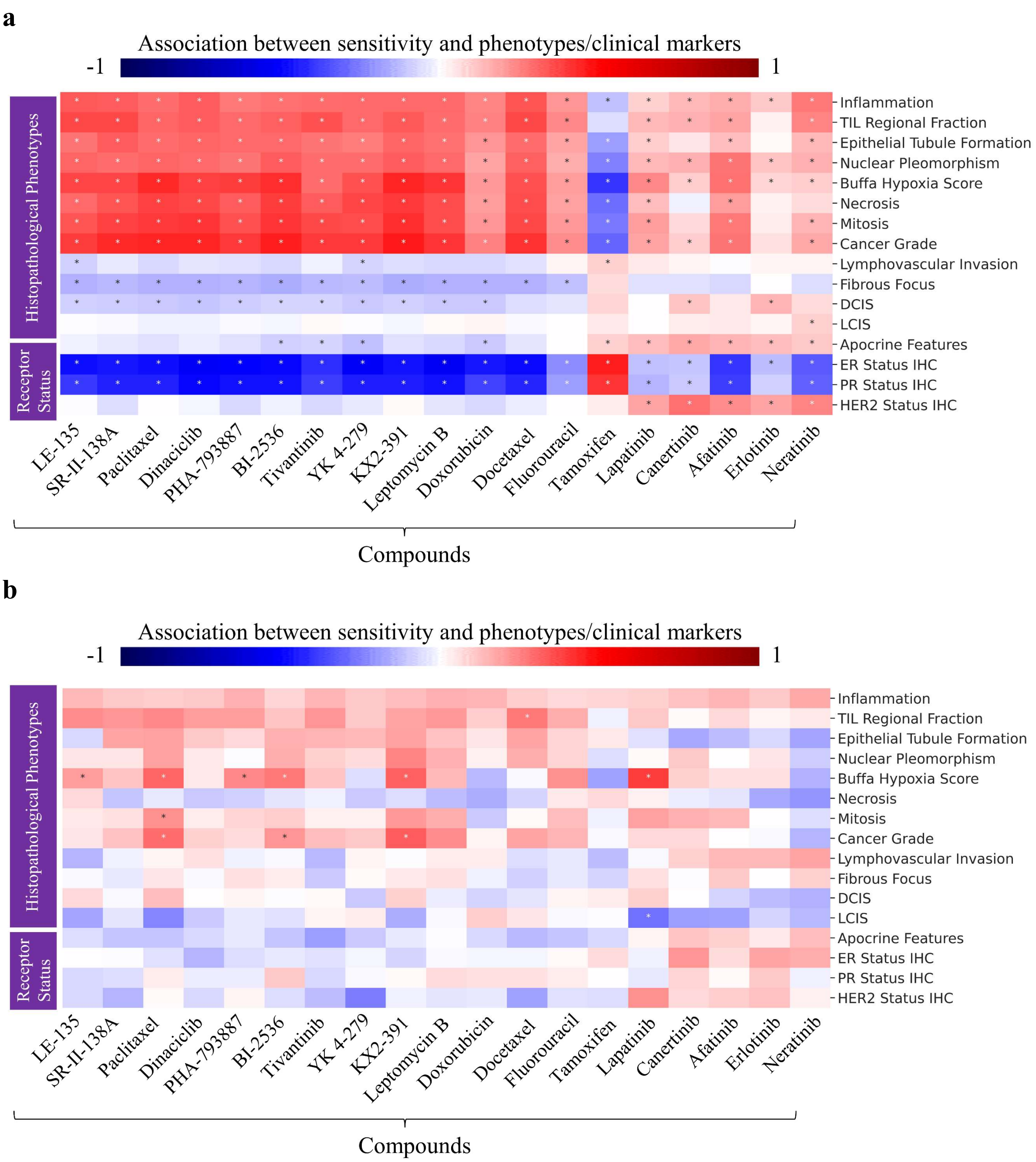

Supplementary Figure 3: Association of gene expression based imputed sensitivity of compounds with pathologist assigned WSI-level histological phenotypes and breast cancer receptor status. Compounds are shown along x-axis, and histological phenotypes and receptor status are shown along y-axis. Red and blue colours indicate the degree of association (Kendall's tau correlation) between patients' gene expression based imputed sensitivity of compounds and a specific histopathological phenotype or clinical marker. Bluer colour shows strong negative correlation while strong positive correlation is shown using dark-red colour. Boxes in the heatmap marked with an asterisk (*) indicate a statistically significant association (determined by the Wilcoxon rank-sum test, $p \ll 0.05$). Figure (**a**) shows the association for patients with Invasive Ductal Carcinoma (IDC), while Figure (**b**) for patients Invasive Lobular Carcinoma (ILC).

Abbreviation: TIL: Tumour Infiltrating Lymphocytes, LCIS: Lobular Carcinoma in situ, DCIS: Ductal Carcinoma in situ, Estrogen Receptor (ER), Progesterone Receptor (PR), Human Epidermal growth factor Receptor 2 (HER2).

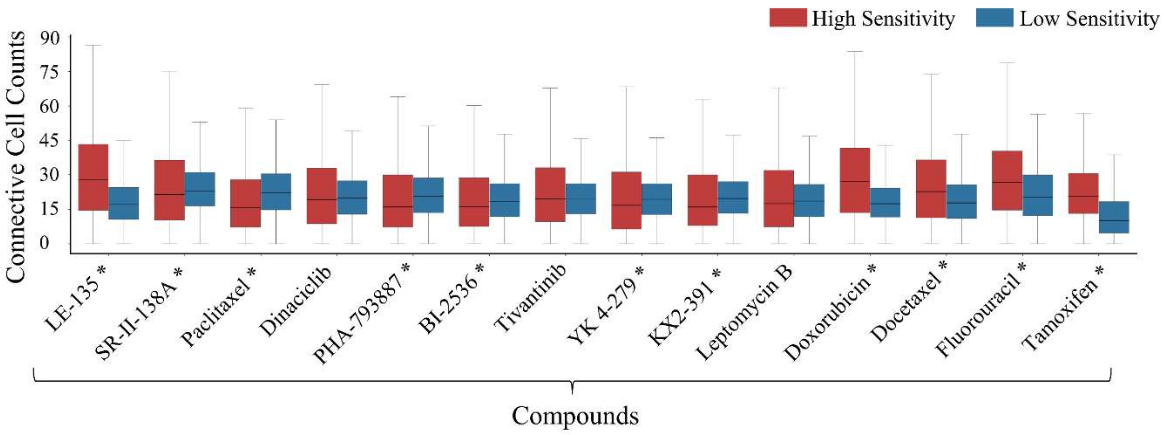

Supplementary Figure S4: Statistical plots showing the association compounds sensitivity with connective cell counts. Compounds are shown along x-axis while the distribution of connective cells is shown along y-axis. Red and blue colour represent the high and low sensitive group, respectively. Compounds with * next to their name show statistically significant (Wilcoxon rank-sum test $p \ll 0.05$) difference in connective cell counts in highly scoring patches of sensitivity and insensitivity.

Supplementary Tables

Supplementary Table 1: List of compounds and their associated histological patterns of high and low sensitivity are shown. The first column lists the name of the compound (CPD), followed by the gene name of the target protein, the compound's target activity, and its approval status (whether it is FDA-approved, in a clinical trial phase, or experimental/probe) all separated by vertical bar (|). Second column displays visual patterns linked to high sensitivity, while the third column showcases those associated with low compound sensitivity. The bar plot below each visual pattern, shows the relative proportions of different cell types present in the patch, while the numbers next to the plot show number of mitotic counts overall cellular counts.

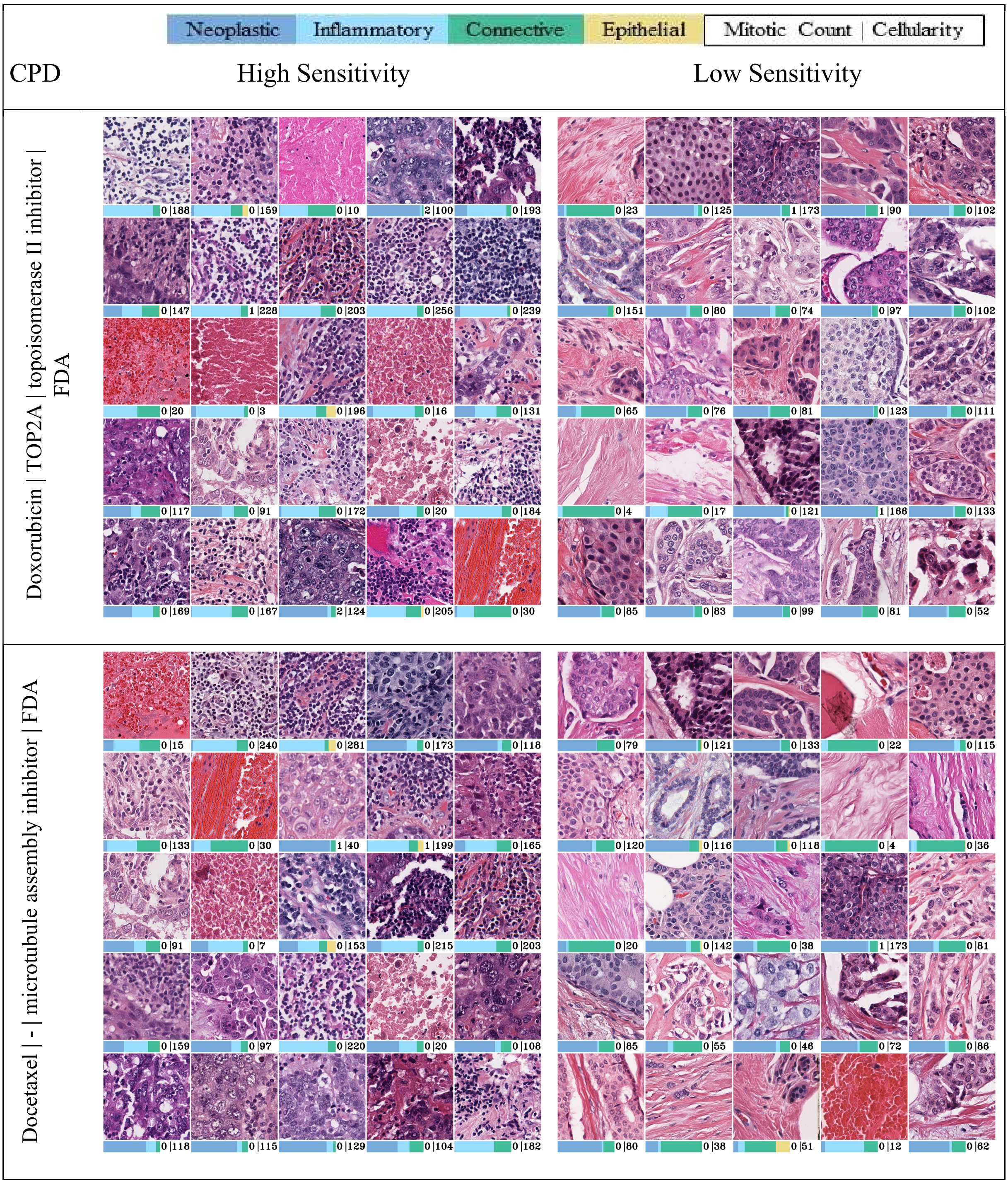

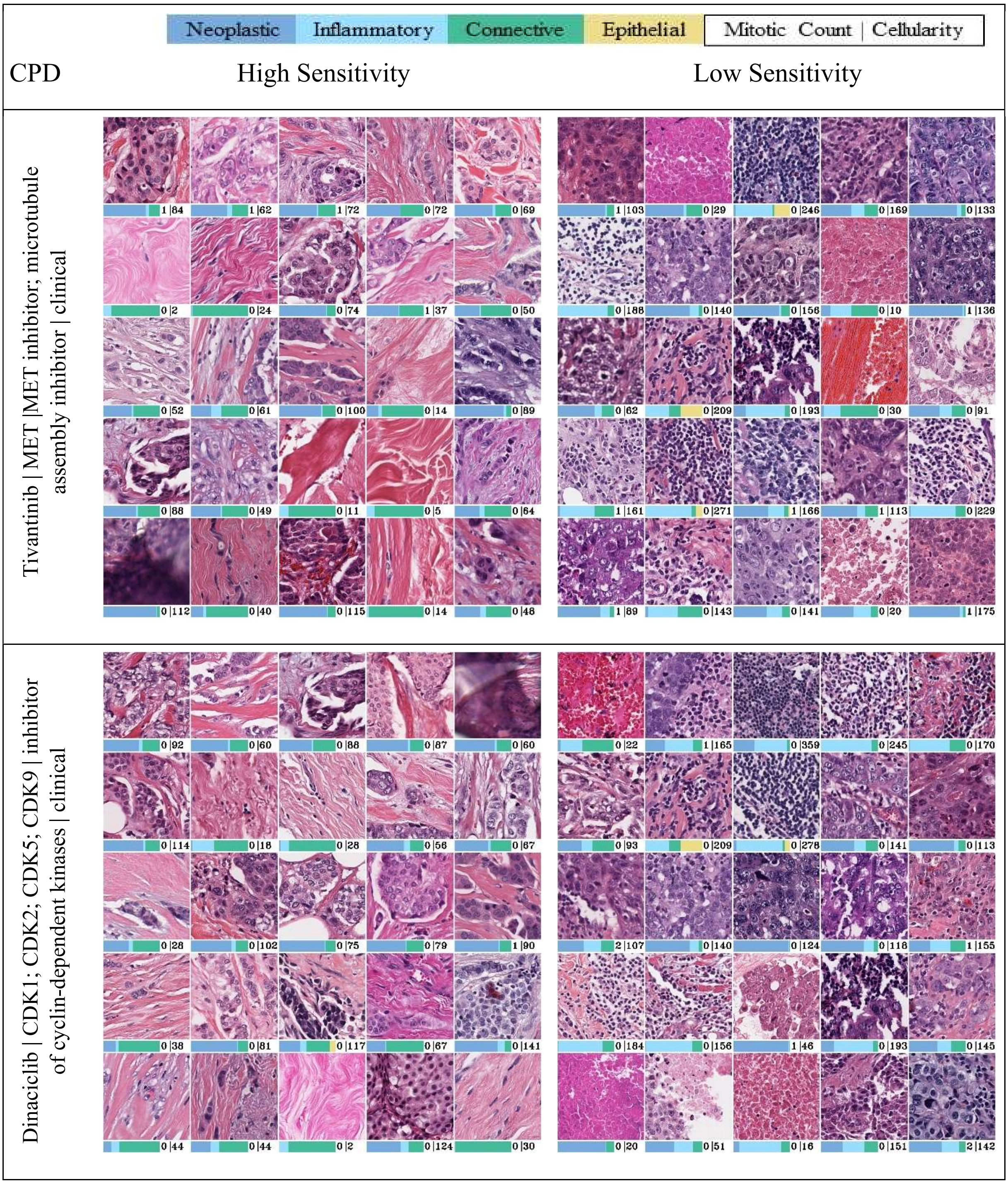
Neoplastic
Inflammatory
Connective
Epithelial
Mitotic Count | Cellularity
CPD
High Sensitivity
Low Sensitivity
Tivantinib | MET |MET inhibitor; microtubule assembly inhibitor | clinical
Dinaciclib | CDK1; CDK2; CDK5; CDK9 | inhibitor of cyclin-dependent kinases | clinical

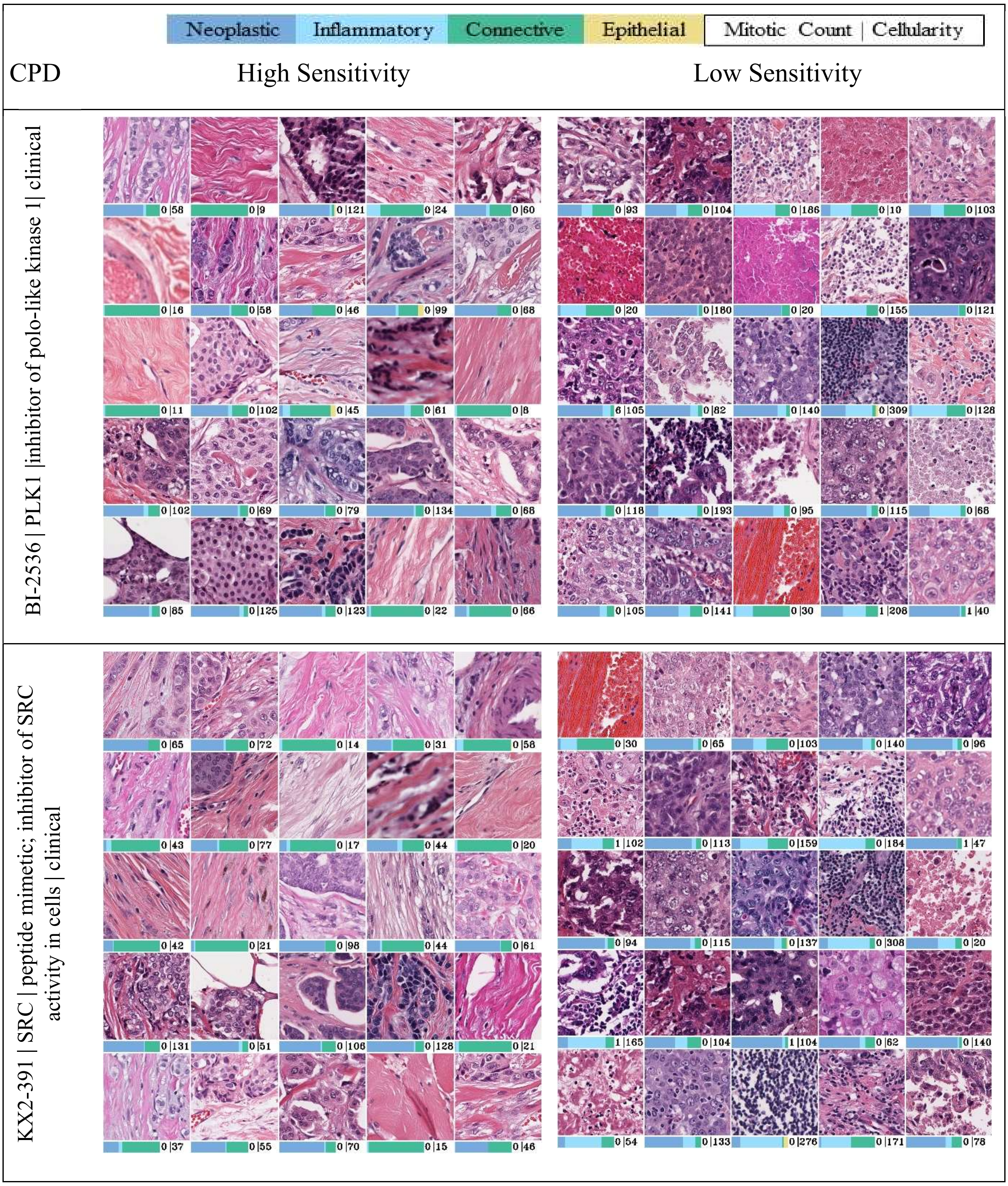
Neoplastic
Inflammatory
Connective
Epithelial
Mitotic Count | Cellularity
CPD
High Sensitivity
Low Sensitivity
BI-2536 | PLK1 |inhibitor of polo-like kinase 1| clinical
KX2-391 | SRC | peptide mimetic; inhibitor of SRC activity in cells | clinical

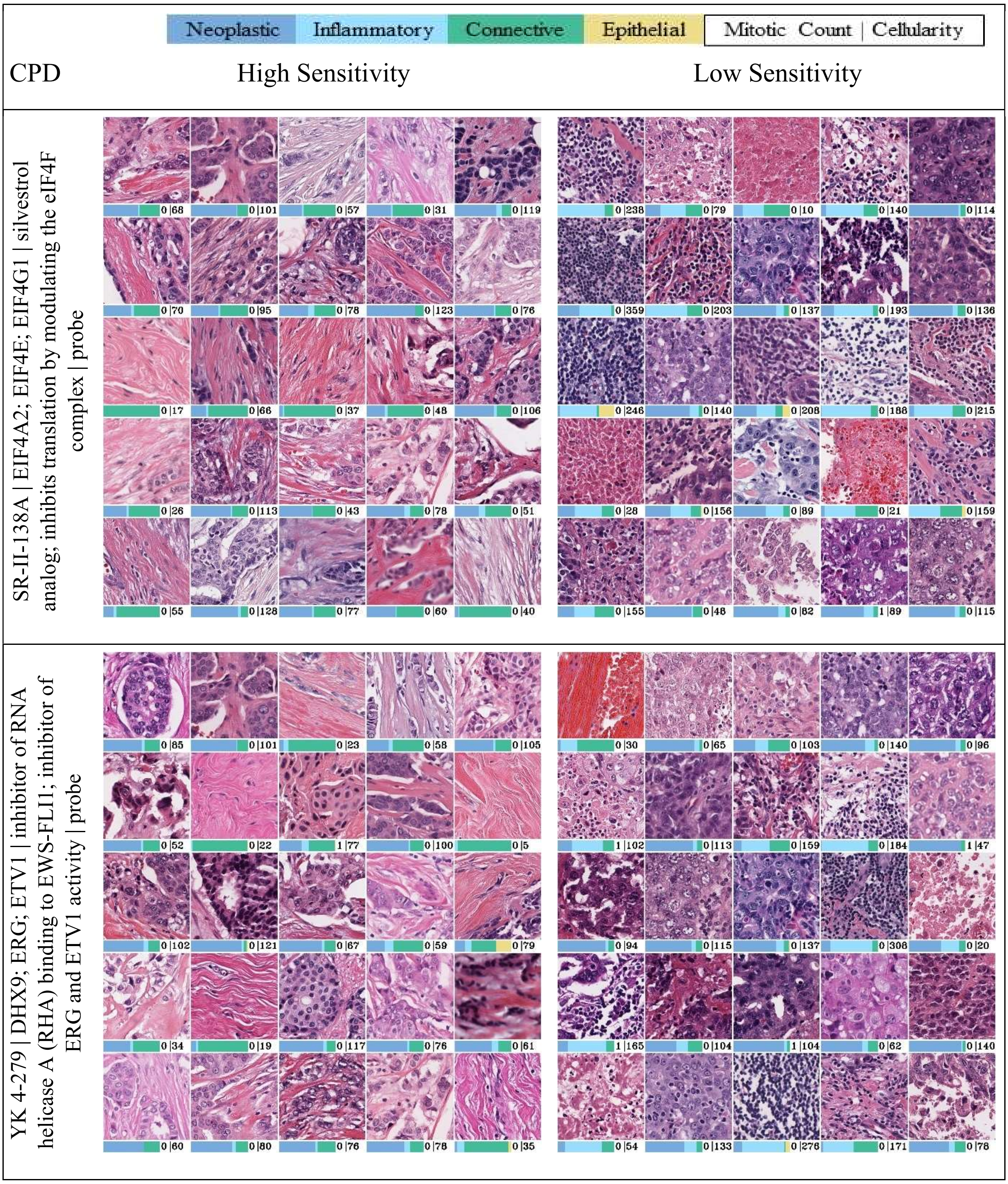
Neoplastic
Inflammatory
Connective
Epithelial
Mitotic Count | Cellularity
CPD
High Sensitivity
Low Sensitivity
SR-II-138A | EIF4A2; EIF4E; EIF4G1 | silvestrol analog; inhibits translation by modulating the eIF4F complex | probe
YK 4-279 | DHX9; ERG; ETV1 | inhibitor of RNA helicase A (RHA) binding to EWS-FLI1; inhibitor of ERG and ETV1 activity | probe

Supplementary Table 2: Association of INCCR (inflammatory to neoplastic cell counts ratio), mitotic counts, and their combined effect (INCCR × Mitotic counts) on patients' sensitivity to compounds. The first column lists compound names, followed by Pseduo-R^2 value indicating the logistic regression model goodness of fit. The last three columns list the model learned coefficient (w), FDR-corrected p-value (p), and 2.5% to 97.5% confidence interval (CI) for features (INCCR, Mitotic counts, Interaction) used in fitting the logistic regression model. The interaction column lists the multiplicative effect of patch-level mitotic counts and INCCR on patients' sensitivity to compounds.

Compounds	Psuedo-R^2	INCCR			Mitotic counts			Interaction		
		w	p	CI (2.5% – 97.5%)	w	p	CI (2.5% – 97.5%)	w	p	CI (2.5% – 97.5%)
**SR-II-138A**	0.24	1.38	0.00	(1.27 – 1.48)	1.39	0.00	(1.13 – 1.68)	0.54	0.00	(0.21 – 0.87)
**Paclitaxel**	0.07	0.36	0.00	(0.26 – 0.46)	1.44	0.00	(1.17 – 1.71)	0.59	0.00	(0.29 – 0.90)
**Dinaciclib**	0.09	0.73	0.00	(0.66 – 0.80)	0.85	0.00	(0.69 – 1.02)	0.35	0.00	(0.15 – 0.54)
**PHA-793887**	0.13	0.83	0.00	(0.76 – 0.92)	1.06	0.00	(0.88 – 1.24)	0.33	0.00	(0.10 – 0.56)
**BI-2536**	0.08	0.66	0.00	(0.55 – 0.77)	1.38	0.00	(1.11 – 1.65)	0.79	0.00	(0.46 – 1.13)
**Tivantinib**	0.09	0.79	0.00	(0.71 – 0.88)	1.17	0.00	(0.95 – 1.39)	0.65	0.00	(0.39 – 0.92)
**YK 4-279**	0.12	0.91	0.00	(0.84 – 0.98)	0.75	0.00	(0.59 – 0.89)	0.28	0.00	(0.09 – 0.47)
**KX2-391**	0.07	0.65	0.00	(0.50 – 0.80)	1.83	0.00	(1.43 – 2.22)	1.20	0.00	(0.74 – 1.66)
**Leptomycin B**	0.13	0.96	0.00	(0.89 – 1.04)	0.83	0.00	(0.67 – 0.99)	0.38	0.00	(0.17 – 0.58)
**Docetaxel**	0.15	1.11	0.00	(1.02 – 1.21)	1.29	0.00	(1.01 – 1.58)	0.71	0.00	(0.41 – 1.02)
**Fluorouracil**	0.13	0.92	0.00	(0.87 – 0.98)	0.56	0.00	(0.44 –0.68)	0.15	0.02	(0.03 – 0.27)
**Doxorubicin**	0.42	2.07	0.00	(1.99 – 2.16)	0.41	0.00	(0.28 – 0.53)	0.16	0.04	(0.01 – 0.31)
**Tamoxifen**	0.02	-0.13	0.00	(-0.18 – -0.08)	-0.36	0.00	(-0.44 – -0.27)	0.039	0.49	(-0.06 – 0.14)
**LE-135**	0.27	1.47	0.00	(1.41 – 1.53)	0.35	0.00	(0.25 – 0.45)	0.01	0.83	(-0.09 – 0.12)
