# Supplementary material for: Cancer drug sensitivity prediction from routine histology images
Source: NPJ Precis Oncol. 2024 Jan 6;8:5. doi: 10.1038/s41698-023-00491-9 (PMC10771481; doi:10.1038/s41698-023-00491-9)
Supplement: Supplementary file 5 — Reporting summary [file 41698_2023_491_MOESM5_ESM.pdf]

nature portfolio

Corresponding author(s): DBPR; 03972-T

Last updated by author(s): Nov 13, 2023

Reporting Summary

Nature Portfolio wishes to improve the reproducibility of the work that we publish. This form provides structure for consistency and transparency in reporting. For further information on Nature Portfolio policies, see our Editorial Policies and the Editorial Policy Checklist.

Statistics

For all statistical analyses, confirm that the following items are present in the figure legend, table legend, main text, or Methods section.

n/a	Confirmed	
☐	☒	The exact sample size (*n*) for each experimental group/condition, given as a discrete number and unit of measurement
☐	☒	A statement on whether measurements were taken from distinct samples or whether the same sample was measured repeatedly
☐	☒	The statistical test(s) used AND whether they are one- or two-sided<br>*Only common tests should be described solely by name; describe more complex techniques in the Methods section.*
☐	☒	A description of all covariates tested
☐	☒	A description of any assumptions or corrections, such as tests of normality and adjustment for multiple comparisons
☐	☒	A full description of the statistical parameters including central tendency (e.g. means) or other basic estimates (e.g. regression coefficient) AND variation (e.g. standard deviation) or associated estimates of uncertainty (e.g. confidence intervals)
☐	☒	For null hypothesis testing, the test statistic (e.g. *F*, *t*, *r*) with confidence intervals, effect sizes, degrees of freedom and *P* value noted<br>*Give P values as exact values whenever suitable.*
☒	☐	For Bayesian analysis, information on the choice of priors and Markov chain Monte Carlo settings
☐	☒	For hierarchical and complex designs, identification of the appropriate level for tests and full reporting of outcomes
☐	☒	Estimates of effect sizes (e.g. Cohen's *d*, Pearson's *r*), indicating how they were calculated

Our web collection on statistics for biologists contains articles on many of the points above.

Software and code

Policy information about availability of computer code

Data collection	Whole slides images (WSIs) of all TCGA-BRCA patients used in the study can be downloaded from the NIH Genomic Data Common Portal at this link: https://portal.gdc.cancer.gov/. For downloading TCGA WSIs we used gdc_client.
Data analysis	tiatoolbox https://github.com/TissueImageAnalytics/tiatoolbox/ v1.4<br>ALBRT https://github.com/engrodawood/ALBRT v1.4<br>torch geometric https://pytorch-geometric.readthedocs.io/en/latest/install/installation.html v2.2<br>PyTorch (2.0) https://pytorch.org/ RRID:SCR_018536<br>Scipy (1.6.2) http://www.scipy.org/ RRID:SCR_008058<br>NumPy (1.23.5) http://www.numpy.org/ RRID:SCR_008633<br>Pandas (1.5.3) https://pandas.pydata.org/ RRID:SCR_018214<br>Matplotlib (3.3.4) https://matplotlib.org/ RRID:SCR_008624

For manuscripts utilizing custom algorithms or software that are central to the research but not yet described in published literature, software must be made available to editors and reviewers. We strongly encourage code deposition in a community repository (e.g. GitHub). See the Nature Portfolio guidelines for submitting code & software for further information.

Data

Policy information about availability of data

All manuscripts must include a data availability statement. This statement should provide the following information, where applicable:

- Accession codes, unique identifiers, or web links for publicly available datasets
- A description of any restrictions on data availability
- For clinical datasets or third party data, please ensure that the statement adheres to our policy

Whole slides images (WSIs) of all TCGA-BRCA patients used in the study can be downloaded from the NIH Genomic Data Common Portal at this link: https://portal.gdc.cancer.gov/. The gene expression based imputed drug response data for the analyzed patients are provided in the paper supplementary materials.

Research involving human participants, their data, or biological material

Policy information about studies with human participants or human data. See also policy information about sex, gender (identity/presentation), and sexual orientation and race, ethnicity and racism.

Reporting on sex and gender	Not applicable
Reporting on race, ethnicity, or other socially relevant groupings	Not applicable
Population characteristics	Not applicable
Recruitment	We do not recruit any participants, but analyzed whole slide images of TCGA breast cancer cohort.
Ethics oversight	Biomedical and Scientific Research Ethics Committee (BSREC) University of Warwick Approved the study under application ID BSREC 16/21-22.

Note that full information on the approval of the study protocol must also be provided in the manuscript.

Field-specific reporting

Please select the one below that is the best fit for your research. If you are not sure, read the appropriate sections before making your selection.

☒ Life sciences ☐ Behavioural & social sciences ☐ Ecological, evolutionary & environmental sciences

For a reference copy of the document with all sections, see nature.com/documents/nr-reporting-summary-flat.pdf

Life sciences study design

All studies must disclose on these points even when the disclosure is negative.

Sample size	The sample size of (n=936 whole slide images) for cancer drugs sensitivity prediction was selected from TCGA. The gene expression based imputed sensitivity for these patients were used from a previous study (Gruner et al., 2021).
Data exclusions	We excluded Whole Slide Images with missing meta data. The exclusion criteria is clearly explained in the manuscript.
Replication	The results reported in the manuscript can be reproduced easily using paper Github rep: https://github.com/engrodawood/HiDS. For model was trained and evaluated using 5-fold cross-validation. The predicted sensitivity of patients to different drugs is provided in the supplementary materials.
Randomization	Randomization of sample for cross-validation folds (5-folds) was performed at random without stratification. The model is trained on four folds and the performance is evaluated on the held out fold.
Blinding	Data of all patients were analyzed anonymously. Not additional blinding was done.

Reporting for specific materials, systems and methods

We require information from authors about some types of materials, experimental systems and methods used in many studies. Here, indicate whether each material, system or method listed is relevant to your study. If you are not sure if a list item applies to your research, read the appropriate section before selecting a response.

Materials & experimental systems

n/a	Involved in the study	
☒	☐	Antibodies
☒	☐	Eukaryotic cell lines
☒	☐	Palaeontology and archaeology
☒	☐	Animals and other organisms
☐	☒	Clinical data
☒	☐	Dual use research of concern
☒	☐	Plants

Methods

n/a	Involved in the study	
☒	☐	ChIP-seq
☒	☐	Flow cytometry
☒	☐	MRI-based neuroimaging

Clinical data

Policy information about clinical studies
All manuscripts should comply with the ICMJE guidelines for publication of clinical research and a completed CONSORT checklist must be included with all submissions.

Clinical trial registration	Not applicable
Study protocol	Not applicable
Data collection	Not applicable
Outcomes	Not applicable

Plants

Seed stocks	Not relevant
Novel plant genotypes	Not relevant
Authentication	Not relevant
